# Distribution, Sources and Water Quality Evaluation of the Riverine Solutes: A Case Study in the Lancangjiang River Basin, Tibetan Plateau

**DOI:** 10.3390/ijerph16234670

**Published:** 2019-11-23

**Authors:** Jinke Liu, Guilin Han, Man Liu, Jie Zeng, Bin Liang, Rui Qu

**Affiliations:** Institute of Earth Sciences, China University of Geosciences (Beijing), Beijing 100083, China; Liujinke@cugb.edu.cn (J.L.); lman@cugb.edu.cn (M.L.); zengjie@cugb.edu.cn (J.Z.); liangbin@cugb.edu.cn (B.L.); Qurui@cugb.edu.cn (R.Q.)

**Keywords:** hydro-geochemistry, major ions, water quality, Himalayan rivers, southwest China

## Abstract

To examine the chemical composition, potential sources of solutes, and water quality of Lancangjiang River, the concentrations of major ions (Ca^2+^, Mg^2+^, Na^+^, K^+^, HCO_3_^−^, SO_4_^2−^, Cl^−^ and NO_3_^−^) in 45 river water samples collected in July and August 2019 were determined. Ca^2+^ and HCO_3_^−^ are the predominant ions in river water. The extremely low K^+^ and NO_3_^−^ concentrations and the sparse population suggest that the anthropogenic inputs are limited. The Pearson correlation coefficients and the elemental ratios Ca^2+^/Na^+^ versus Mg^2+^/Na^+^, Ca^2+^/Na versus HCO_3_^−^/Na^+^, [Ca^2+^ + Mg^2+^]/[HCO_3_^−^] versus [SO_4_^2−^]/[HCO_3_^−^] reveal the mixing processes of different sources; the chemical composition of the river water is controlled by the mixture of carbonate weathering, evaporite weathering and silicate weathering inputs. To quantify the contributions of atmospheric input and rock dissolution, the forward method is employed in this study, which is based on the mass balance equation. The calculation results suggest the carbonate weathering inputs and gypsum dissolution make up the majority of the riverine cations, while silicate weathering and halite dissolution constitutes a relatively small proportion, the contributions of the atmospheric input are limited. The fast dissolution rate of evaporite and carbonate minerals and their lithologic distributions should be the key factor. To evaluate the water quality for drinking and irrigation purposes, the drinking water quality guidelines and the calculated parameters were employed, including sodium adsorption ratio (SAR), soluble sodium percentage (Na%,) and residual sodium carbonate (RSC). The assessments indicate that the river waters in the middle-lower reaches are generally suitable for irrigation and drinking purpose, and will not lead to health and soil problems, such as soil compaction and salinization. While in the upper reaches, the dissolution of carbonate and gypsum minerals transport abundant ions into river water and the river waters are not appropriate to use directly. This result highlights that the water quality status can also be affected by natural weathering processes in the area without anthropogenic inputs, where the long-time monitoring of water quality is also necessary.

## 1. Introduction

River water is the important component of hydrosphere and draws great attention in geological and environmental research, for it constitutes the lateral channels transporting the territorial matters towards the ocean [1,2]. The riverine dissolved loads transported along river waters are derived from both natural processes and anthropogenic perturbations [3,4,5,6], among which the chemical weathering is considered as the dominant source [1,7,8]. Thus, estimating the contributions of the different minerals during chemical weathering to the riverine solutes can help researchers to better understand the chemical composition of river water and regular patterns of matter cycling [9,10]. The forward method based on the mass balance equations is a common and effective way to estimate the contributions of different sources [8,11,12,13]. Atmospheric inputs, silicate weathering, carbonate weathering, and evaporite dissolution can be determined by assuming the elemental ratios between the major ions.

As the water supplies for drinking, agricultural activities and industry, the river water, especially water quality gains wide public concern over the world [14,15], the concentrated solutes may not only result in serious environmental problems (e.g. eutrophication) [16], but also threaten the daily life of local residents [17,18]. The inappropriate water with high salinity and alkalinity used for irrigation may destroy soil aggregate structure, thus reduce the grain yields [19,20,21], additionally, drinking water with deleterious or toxic matters may cause health hazards for the residents [22,23]. 

Several studies have found that the natural weathering processes which transport abundant ions into the river water can significantly affect river water quality in the area with little anthropogenic perturbations [24,25,26]. The Lancangjiang River basin is a part of eastern Himalayan region and the Qinghai–Tibet Plateau (HQTP). As the Water Tower of Asia, the HQTP is characterized by the high elevation and little human activity [27,28]. Plenty of research has been done about the eastern Himalayan river system [13,27,28,29,30,31]; these previous studies focused on the weathering rates and CO_2_ consumption flux, because the CO_2_ sequestration during the chemical weathering is considered as the sink of atmospheric CO_2_ levels, and the Himalayan lift significantly increase the chemical weathering rates, which is a major driver of global climate change [32]. However, few studies have examined the water quality in Himalayan river system. As the abundant solutes derived from the weathering may cause environmental problems, a comprehensive water quality assessment to examine the environmental impact of the dissolved load is necessary. The main aims of this study are: (1) to examine the chemical composition of the Lancangjiang River water; (2) to distinguish the sources of the solutes and estimate their relative contributions; (3) to assess the Lancangjiang River water quality status. Knowledge of the hydrogeochemistry and water quality status can help us to better understand the geochemical processes to improve environmental management in the Lancangjiang River basin.

## 2. Materials and Methods 

### 2.1. Study Area Description and Sample Collection

**Figure 1:**
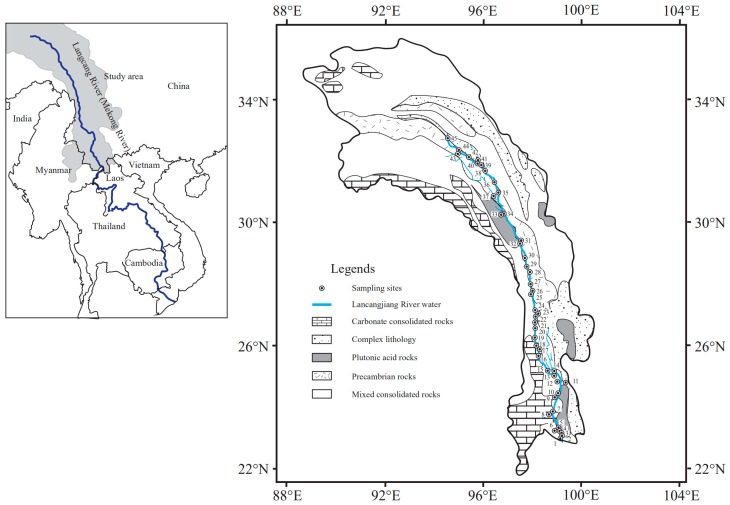
Sketch map showing the sampling sites, locations, lithology and river network of Lancangjiang River.

The study area is located in the eastern margin of the Tibetan Plateau, the collision of the Eurasian plates and Indian plates forged the steep valleys topography and elongated pattern of lithology in HQTP [33,34]. As showed in Figure 1, the lithology can be divided into several groups [35]: (1) carbonate consolidated rocks, including limestone, dolomite, and chalk, which make up more than 80% of the rock in study area; (2) complex lithology, encompassing sedimentary, volcanic rocks, metamorphic rocks and evaporites; (3) Plutonic acid rocks, including granite and diorite; (4) Precambrian rocks consisting of medium-highly granodioritic-granitic metamorphic rocks; (5) mixed consolidated rocks, typically sediments with 30%–70% carbonate minerals. The predominant minerals evaporites are halite and gypsum, which are scattered across the whole study area. Overall, the carbonate minerals make up approximately 65% of the lithologic composition in the study area, with silicate at 30% and evaporate at 5% [28].

Lancangjiang River (33°81′N–21°75′N and 94°40′E–101°15′E), which is the upper-middle reaches of Mekong River, originates from Tanggula Mountain with a drainage area of 167,400 km^2^, a length of 2160 km in China, and a mean annual discharge of 60 km^3^ per year [33]. The source area is located in the HQTP with a mean elevation of about 4500 m. In the upper reaches (Qinghai and Tibet province), the topography is characterized by high elevation (>3000 m) and deep valleys, the vegetation covering type is alpine steppe; while in lower reaches (Yunnan province), the vegetation covering type is subtropical forest, coniferous forest, and scrub meadow [13]. The Lancangjiang River Basin has a monsoon climate [29], leading to high rainfall in summer (>60% of the annual precipitation) with a mean annual precipitation of about 560 mm [13]. The mean air temperature is from −7 to 16 °C, with an average of 1 °C [28]. The human perturbations are considered limited because of the relatively scarce population compared to other provinces in China [29].

### 2.2. Sample Collection and Analysis

Forty-five river water samples were collected in Lancangjiang River in July and August in 2019: 30 samples were collected in mainstream, 12 samples in tributaries, 2 in the source area and 1 from industrial sewage, respectively. The location information of the sampling sites is presented in Figure 1 and Table S1. All the river samples were collected under a depth ~0.2 m from the center of the river on bridges or ferries. The collected river waters were filtered through 0.22-μm Millipore membrane (Whatman GF/F, pre-cleaned, General Electric Company(GE), Boston, MA, USA) within 24 h, then stored in precleaned polyethylene bottles. The river samples for cations measurement were acidified with 0.02 M HCl to pH < 2. All the containers were sealed and kept refrigerated at about 4 ℃ until further analysis.

The physical-chemical parameters of Lancangjiang River, including water temperature (T), pH, and electrical conductivity (EC) were immediately measured in the field by YSI-6920 (Xylem Inc., Yellow Springs, OH, USA). TA (total alkalinity) was titrated by HCl (0.03 M) in the field. The major anions (Cl^−^, NO_3_^−^, SO_4_^2−^) were measured by ion chromatograph ICS-900 (DIONEX., Sunnyvale, CA, USA) in China University of Geoscience, Beijing (CUGB). Cations (Ca^2+^, Mg^2+^, Na^+^ and K^+^) were measured by ICP-OES (Optima 5300DV, PerkinElmer Inc., Waltham, MA, USA) in the Institute of Geographic Sciences and Nature Resources Research, Chinese Academy of Sciences (CAS). Replicate samples were measured to guarantee the accuracy of all the analysis, the relative standard deviations of all the analyses were within ± 5%. The results of analyses are presented in Table S1.

The correlation analyses are conducted by SPSS 19.0 software (SPSS Inc., Chicago, IL, USA) and the figures were completed with Origin 2018 (Origin Lab., Hampton, Massachusetts, MA, USA) and Adobe Illustrator (Adobe Inc., San Jose, CA, USA). The inorganic carbon species including HCO_3_^−^, CO_3_^2−^, H_2_CO_3_, and aqueous CO_2_ are calculated by using the program CO2SYS [36].

### 2.3. Mass Balance Model Calculation

In order to quantify the relative contributions of different sources, the forward method based on mass balance was employed [13]. The assumed mass balance equations can be written as Equations (1–6):

[Cl^−^]_riv_ = [Cl^−^]_atm_ + [Cl^−^]_evap_ (1)

[SO_4_^2−^]_riv_ = [SO_4_^2−^]_rain_+ [SO_4_^2−^]_evap_ (2)

[Na^+^]_riv_ =[Na^+^]_rain_ + [Na^+^] _evap_ + [Na^+^]_sil_ (3)

[K^+^]_riv_ =[K^+^]_rain_ + [K^+^]_sil_ (4)

[Ca^2+^]_riv_ =[Ca^2+^]_rain_ + [Ca^2+^] _evap_ + [Ca^2+^]_sil_ + [Ca^2+^]_carb_ (5)

**Figure 2:**
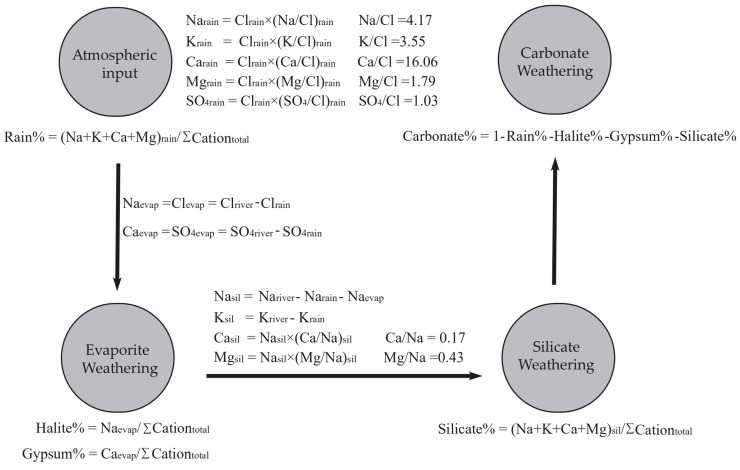
The outline of forward method calculation used in this study.

[Mg^2+^]_riv_ =[Mg^2+^]_rain_ + [Mg^2+^]_sil_ + [Mg^2+^]_carb_ (6)

where riv = river; sil = silicate; carb = carbonate; evap = evaporite, respectively. The procedure of calculating the contributions of these sources are shown in Figure 2.

First of all, the contributions of atmospheric inputs can be determined by the Cl^−^ concentrations. Because Cl^−^ is conservative in river, the Cl^−^ contents in river water derived from rain should keep the same level. The widely accepted method is assuming that the sample with the lowest Cl^−^ concentration acquired all its dissolved Cl^−^ completely from the atmospheric inputs, thus other ions derived from the rain can be calculated by the elemental ratio in rain [5,6,7,8,9,12,13,14]:

[X]_rain_ = [Cl]_lowest_ × (X/Cl)_rain_ (7)

where [X]_rain_ is the element X concentrations in rain waters, [Cl]_lowest_ is the concentrations of Cl^−^ which is assumed completely from the precipitation, (X/Cl)_atm_ is the X/Cl^−^ molar ratio in rain waters. The sample LCJ-25 (Langcangjiang River-25) with the lowest Cl^−^ concentration (3.5 μmol/L) is collected in the tributaries in pristine areas, the bedrock in the sampling site LCJ-25 is Precambrian rocks consisting of medium-highly granodioritic-granitic metamorphic rocks, in which the chloride contents are limited.). These values is similar to the concentrations in rain in this study area (3.3 μmol/L [28], 2.9 μmol/L [37], respectively). In this study, the rain samples were not collected; the (X/Cl)_atm_ used here are the long-time average values as reported in the previous study [37]. Then, the contributions of evaporite can be determined by assuming the Cl^−^ remaining after the atmospheric input correction are from halite, and all SO_4_^2−^ comes from gypsum dissolution. It is noteworthy that the estimated gypsum contributions here involve the sulfide, consequently, these contributions are the maximum limit of evaporite dissolution. Next, for the silicate fraction, all Na^+^ remaining after atmospheric input and evaporite dissolution while all K^+^ remaining after atmospheric input are assumed from silicate weathering. The predominant evaporites are halite and gypsum, the contributions of evaporites to riverine K^+^ are negligible. To estimate the contributions of silicate weathering to the riverine Mg^2+^, Ca^2+^, the molar ratios of Mg^2+^/Na^+^ and Ca^2+^/Na^+^ in the tributaries draining silicate terrains are employed. It is noteworthy that the molar ratios used here should not be that in silicate rocks, because erosion processes may fractionate these ratios. The Mg^2+^/Na^+^ and Ca^2+^/Na^+^ ratios are assumed to be 0.43 and 0.17, respectively (as reported in the previous study [29]). The Mg^2+^ and Ca^2+^ derived from the silicate weathering can be calculated by using these ratios and silicate-derived Na^+^ contents. Finally, the contribution of carbonate weathering can be simply calculated based on the mass balance: 100% - rain inputs% - evaporite weathering% - silicate weathering%, based on the mass balance. The contributions of anthropogenic inputs are limited (discussed in the next section). The results are shown in Table S2.

### 2.4. Water Quality Evaluation

Lancangjiang River is the major water supply (agricultural irrigation and drinking) for the local residents. In order to assess the suitability for irrigation and drinking purposes, the physicochemical parameters EC (electrical conductibility) and ion concentrations of the river waters are evaluated following drinking water quality guidelines (Chinese drinking water standards: GB 5749-2006 and WHO guidelines (WHO 2017)). The quality of irrigation water can affect soil quality attributes and further the crop yields [19,20,21]. The sodium hazard assessment for irrigation purpose is conducted by the calculated parameters including SAR, Na% and RSC. The detailed calculation processes are listed as Equations (8–10) [21]:

SAR = Na^+^×[2/(Ca^2+^+Mg^2+^)]^1/2^ (8)

Na% = 100%×Na^+^/(Na^+^+K^+^+Ca^2+^+Mg^2+^) (9)

RSC = (HCO_3_^−^ + CO_3_^2−^) - (Ca^2+^+Mg^2+^) (10)

where the used ion concentrations are equivalent concentrations (meq/L). The results are shown in Table S2.

## 3. Results and Discussion

### 3.1. Hydrochemical Characteristics of the River Water

The data of physicochemical parameters and major ionic concentrations are shown in Table S1. The EC values varied from 81 to 2016 μS/cm with an average of 440 μS/cm. The river water temperature ranged from 8.4 to 28.6 °C with an average of 18.3 °C; the elevation of the sampling sites changes from 4604 m in the sources area to 527 m in the lower reaches, the variation of river water temperature reflects the elevations change. The pH ranged from 7.7 to 8.7 with an average of 8.3, indicating river waters are slightly alkaline. The pH value and water temperature predominate the form of dissolved inorganic carbon species, namely, the relative proportion of HCO_3_^−^, CO_3_^2−^, H_2_CO_3_, and aqueous CO_2_ [38], the concentrations of each proportion can be calculated by using TA, pH, and water temperature, the calculated results suggest that the bicarbonates (HCO_3_^−^) make up more than 95% of the DIC (dissolved inorganic carbon); thus, in this study, the DIC is replaced by the HCO_3_^−^ in the discussion section.

**Figure 3:**
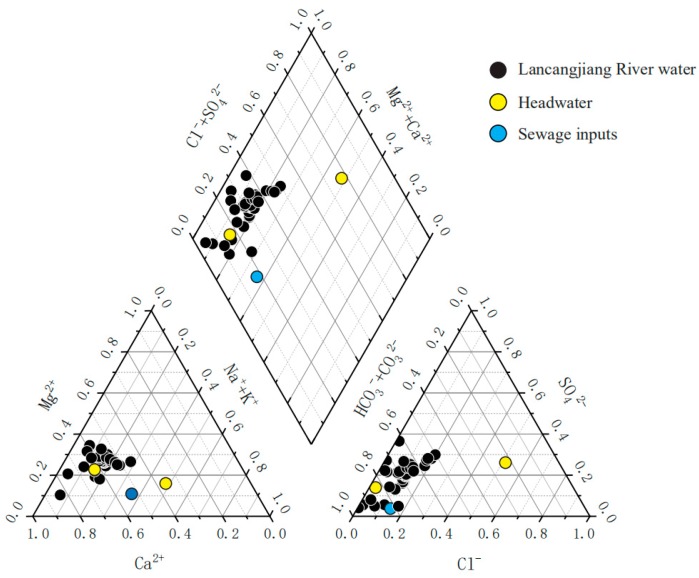
Piper diagrams showing the relative proportions of major ions in Lancangjiang River.

The ionic compositions of Lancangjiang River are exhibited as the piper diagrams (Figure 3). Ca^2+^ is the predominant cation in Lancangjiang River, which constitutes more than 50% of the total cations, Mg^2+^ and Na^+^ + K^+^ make up ~25%, respectively. The order of the anion concentrations is HCO_3_^−^ > SO_4_^2−^ > Cl^−^, the NO_3_^−^ concentrations are much lower and make up less than 5% of the total anions. It is remarkable to note that the K^+^ concentrations are also low (<100 µmol/L)—generally, K^+^ and NO_3_^−^ are considered vulnerable to pollution, especially the agricultural activities [25,39]. The low concentrations of K^+^ and NO_3_^−^ suggest that the contributions of the agricultural activities are limited. The ionic absolute concentration of samples collected in industrial sewage (LCJ-9) is similar to it in other river samples, indicating that the anthropogenic inputs are negligible. The Ca^2+^, Mg^2+^, and HCO_3_^−^, which are always unaffected by the pollution, are mainly derived from the CO_2_-related weathering processes [40,41]. The dominance of Ca^2+^, Mg^2+^ and HCO_3_^−^ concentrations suggest that the carbonate/silicate weathering inputs contribute to the bulk of major ionic budgets. The Cl^−^ and SO_4_^2−^ concentrations vary at a relatively large range and the anthropogenic inputs are considered limited in the study area, revealing that there are likely other sources for the riverine Cl^−^ and SO_4_^2−^ except atmospheric inputs, the evaporite dissolution may also make up a fair proportion of the solutes, because of the exposure of evaporite minerals in the Lancangjiang River basin.

### 3.2. Relationship between the Major Ions

The results of the correlation analysis are shown in Table 1. The results show that Ca^2+^ are clearly correlated with Mg^2+^ and HCO_3_^−^ (r = 0.959, *p* < 0.01; r = 0.839, *p* < 0.01, respectively), Mg^2+^ also exhibited significant correlations with HCO_3_^−^ (r = 0.805, *p* < 0.01), indicating they may have the same sources. Na^+^ is significantly correlated with Cl^−^ while Ca^2+^ are significantly correlated with SO_4_^2−^, which demonstrates the contributions of evaporite dissolution. These results reveal that a complex weathering process may occur in the watershed. However, the correlation analysis is disturbed by the dilution effect, namely the correlations observed may reflect the dilution by run-off rather than the source’s signals [7]. Compared to the absolute concentrations, the elemental ratios are the better tools to describe the mixture between different end-members, because the elemental ratios can eliminate dilution effect. The elemental ratios, such as Ca^2+^/Na^+^, Mg^2+^/Na^+^, in river water are partly inherited into rocks, thus the large differences of these ratios between the silicate minerals and carbonate minerals should be reflected in river water if the solutes are the mixture of silicate weathering and carbonate weathering. Similarly, the elemental ratios can reveal the specific mineral’s dissolution according to the stoichiometry. All of these employments are shown in the next section.

### 3.3. Stoichiometry of Weathering Processes

**Figure 4:**
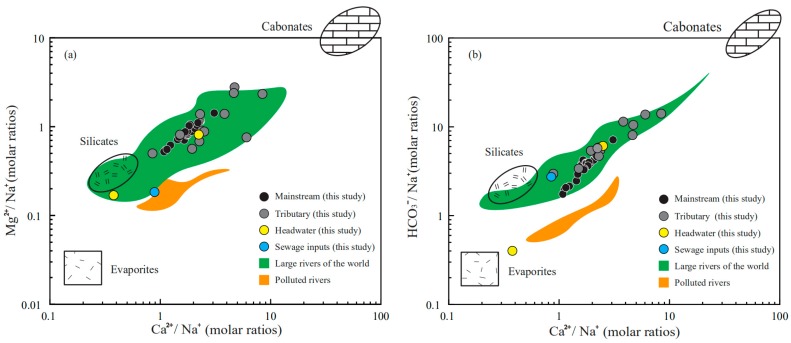
Mixing diagrams using: (**a**) Ca^2+^/Mg^2+^ versus Mg^2+^/Na^+^; (**b**) Ca^2+^/Mg^2+^ versus HCO_3_^−^/Na^+^ molar ratios in the Lancangjiang River, the end-members values and data of large rivers and polluted river are from Gaillardet et al., 1999 [7].

The elemental ratios Ca^2+^/Na^+^ versus Mg^2+^/Na^+^ and Ca^2+^/Na versus HCO_3_^−^/Na^+^ can reflect the mixing of different lithologies [7]; the elemental ratios in river waters inherit that of the minerals. The assumed silicate end-members are Ca^2+^/Na^+^ = 0.35 ± 0.15, Mg^2+^/Na^+^ = 0.24 ± 0.15, HCO_3_^−^/Na^+^ = 2 ± 0.5 while carbonate end-members are Ca^2+^/Na ≈ 50, Mg^2+^/Na^+^ ratios close to 10, HCO_3_^−^/Na^+^ ≈ 50. The end-members used here were reported in previous studies [7]. For the evaporite, the end-members have a wide range and is characterized by the lowest Ca^2+^/Na^+^, Mg^2+^/Na^+^ and HCO_3_^−^/Na^+^ values. As shown in Figure 4, all of the river water samples are distributed between the carbonate end-member and silicate/evaporite end-members, indicating the mixing of the weathering products. The samples collected in the industrial sewage inputs exhibit relatively low Ca^2+^/Na^+^, Mg^2+^/Na^+^ and HCO_3_^−^/Na^+^ values—the most possible cause is that Na^+^ compared Ca^2+^ and Mg^2+^ constitute a relatively large proportion of cations in the sewage inputs [12,25]. The samples collected in the sources area show completely different chemical compositions, the sample LCJ-43 has conspicuously high Na^+^ and Cl^−^ contents, the sampling site is located in the pristine area without human disturbance, and the plot of Ca^2+^/Na versus HCO_3_^−^/Na^+^ is close to the evaporite end-members, proving the large contributions of evaporite dissolutions. However, the ions in sample LCJ-45 are predominated by Ca^2+^, Mg^2+^ and HCO_3_^−^, indicating the mixing of carbonate and silicate weathering. The variations of the chemical compositions in source area are closely related to the complex lithology.

The equivalent ratio between [Ca^2+^ + Mg^2+^]/[HCO_3_^−^] and [SO_4_^2^^−^]/[HCO_3_^−^] can trace the potential weathering processes, which can be summarized as Equations (11–16) [25,42]:

2Ca_x_Mg_(1−x)_CO_3_ + H_2_SO_4_ → 2XCa^2+^ + 2(1 − X)Mg^2+^ + 2HCO_3_^−^ + SO_4_^2−^ (11)

Ca_x_Mg_(1−x)_CO_3_ + H_2_O + CO_2_ → XCa^2+^ + (1 − X)Mg^2+^ + 2HCO_3_^−^ (12)

2Na_x_K_(1−x)_AlSi_3_O_8_ + H_2_SO_4_ → 2Na^+^ + 2(1 − X)K^+^ + SO_4_^2−^ + 6SiO_2_ + 2AlOOH (13)

2Na_x_K_(1−x)_AlSi_3_O_8_ + H_2_CO_3_ → 2Na^+^ + 2(1 − X)K^+^ + 2HCO_3_^−^ + 6SiO_2_ + 2AlOOH (14)

CaAl_2_Si_2_O_8_ + H_2_SO_4_ → Ca^+^ + SO_4_^2−^ + 2SiO_2_ + 2AlOOH (15)

CaAl_2_Si_2_O_8_ + H_2_CO_3_ → Ca^+^ + 2HCO_3_^−^ + 2SiO_2_ + 2AlOOH (16)

**Figure 5:**
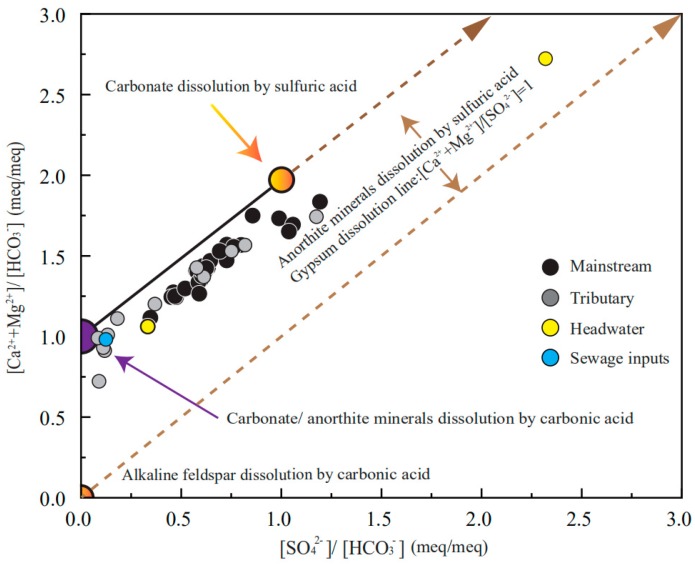
Plots of the major ions: [SO_4_^2−^]/[HCO_3_^−^] versus [Ca^2+^ + Mg^2+^]/[HCO_3_^−^] showing the weathering processes.

According to the reaction equations, several ideal conditions can be obtained: (1) Carbonate are dissolved by the carbonic acid, as the generated HCO_3_^−^ are derived from both the minerals and weathering agents. Thus, the [Ca^2+^ + Mg^2+^]/[HCO_3_^−^] should be equivalent to 1, meanwhile the contributions of H_2_SO_4_ to dissolution should be limited, and [SO_4_^2−^]/[HCO_3_^−^] ratios should be close 0 (Equation 12). In fact, the anorthite weathering by carbonic acid causes the same [Ca^2+^ + Mg^2+^]/[HCO_3_^−^] and [SO_4_^2−^]/[HCO_3_^−^] ratios; the difference is the generated HCO_3_^−^ are completely from the weathering agents (Equation 16); (2) Carbonate minerals are dissolved only by the H_2_SO_4_, the [Ca^2+^ + Mg^2+^]/[HCO_3_^−^] are equal to 2, [SO_4_^2−^]/[HCO_3_^−^] ratios should be close to 0, respectively; (3) The dissolution of alkaline feldspar by the carbonic acid will generate HCO_3_^−^, Na^+^ + K^+^ the [Ca^2+^ + Mg^2+^]/[HCO_3_^−^] and [SO_4_^2−^]/[HCO_3_^−^] ratios are close to 0; (4) The dissolution of anorthite minerals by sulfuric acid and gypsum dissolution will not fall in the specific point; this dissolution leads to [Ca^2+^ + Mg^2+^]/[SO_4_^2−^] = 1—it will just look like a line that goes upward with slope 1. All of this mixing activity should create a linear mixture because of the same denominator. As shown in Figure 5, though the explanation is not unique, the contributions of alkaline feldspar dissolution should be limited, because most samples are far from this end-member. 

There are two potential mixtures between: (1) Carbonate/anorthite dissolved by the carbonic acid and carbonate dissolved by sulfuric acid; (2) Carbonate/anorthite dissolved by the carbonic acid and anorthite minerals dissolved by sulfuric acid/gypsum dissolution. Certainly, the coincidence of these two conditions is also possible; however, in either condition, the contributions of carbonate mineral dissolution to solutes should make up a fair proportion. To further examine the contributions of different lithologies, the forward method is employed in the next discussion.

### 3.4. The Mixing Proportions

**Figure 6:**
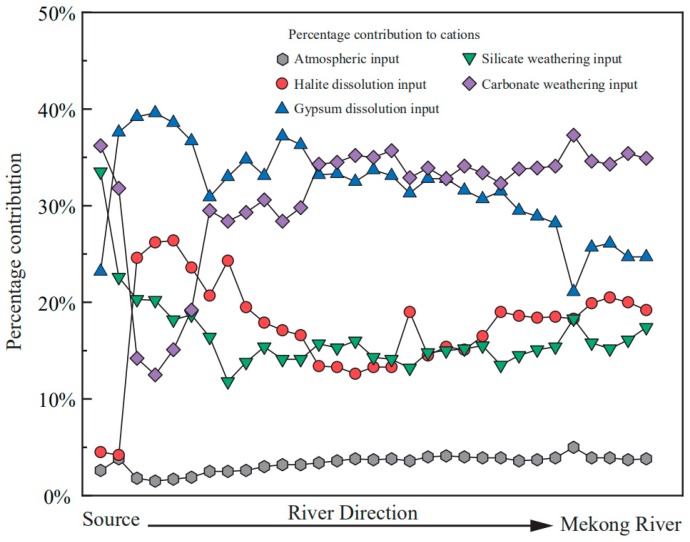
The calculated contributions of different sources to the riverine cations along the mainstream.

The results of the forward method are shown in Table S2, the relative proportions of different sources, including atmospheric input, halite dissolution input, gypsum dissolution input, silicate input and carbonate input are along the mainstream showed in Figure 6. The contributions of the atmospheric inputs are limited, varying from 1.5% to 5% in the mainstream. In some tributaries, the contributions are larger, reaching between 0.6% and 19.3%. The contributions of the halite in the mainstream varies from 4.1% to 26.3%, with an average value of 17.6%, while for that of silicate ranges between 11.7% and 33.5%, averaged 16.4%. The gypsum dissolution inputs make up a mean value of 31.9% of the total cations while the mean contribution of carbonate mineral dissolution is 30.9%. The samples in the last site can better represent the solute sources of the whole basin. In the sample site LCJ-1, 3.8% of the total cations are derived from the rain inputs, while the contributions of the halite dissolution, gypsum dissolution, silicate weathering and carbonate weathering make up 19.2%, 24.7%, 17.4% and 34.9% of the total cations, respectively. Similar to many studies [5,6,7,8,12,13], the carbonate weathering is the predominant sources of the riverine solutes. As mentioned before, the estimate regarding the contribution of gypsum should be the maximum value because this estimated contribution value involves the contribution of sulfate oxidation to the SO_4_^2^^−^. In order to get a better estimate, the data about the S, O isotopic composition in the QHTP rivers were collected [43]. The results suggest that the proportion of sulphate from the sulphate oxidation ranges between 16% and 72%. Thus, the relative proportion of sulphate from gypsum dissolution is at least 28% and the conservative estimate of the gypsum dissolution contribution is about 4% for the whole watershed.

Generally, these relative contributions are dominated by the dissolution rate and lithologic distribution of the watershed [44,45]; the order of dissolution rates (kinetics) is evaporite > carbonate > silicate [44,45,46,47]. Though the evaporite constitutes a small proportion of the bedrock, the fast dissolution rate enables a large proportion of evaporate dissolution. The sample LCJ-43 located in the source area has the largest contributions of halite input, the Na^+^/Cl^−^ ratio is 1.05, indicating that this calculation is reasonable. The predominance of Ca^2+^ in cations is attributed to the mixture of carbonate weathering and gypsum dissolution. The relative fast dissolution rate of these minerals transports abundant Ca^2+^ into the river water. The mixing relationship in Figure 5 can be interpreted by the mixture of carbonate dissolved by the carbonic acid and gypsum dissolution. As discussed previously, the contributions of the gypsum dissolution may be overestimated: the sulfide oxidization will produce sulfuric acid and take part in the rock dissolution processes [48,49], thus the actual contribution of carbonate weathering should be higher. The fluctuation of the contributions along the river direction reflect the lithology discharged by the mainstream and inflows of the tributaries 

### 3.5. Drinking and Irrigation Water Quality Assessments

Lancangjiang River are agricultural, industrial and residential water sources for local residents in both China and Thailand. The drinking water quality guidelines (Chinese drinking water standards: GB 5749-2006 [50] and WHO guidelines [51] are shown in Table 2. The recommended pH varies between 6.5 and 8.5. Most river samples exhibit desirable pH values, however, some samples in the urban area have high pH values, which may result from the anthropogenic inputs. The TDS (total dissolved solids), F, Cl^−^ concentrations are within the respective limits, while the SO_4_^2−^ contents in the head water are close to the limit value, the SO_4_^2−^ concentration of sample LCJ-43 exceeds the recommended values, the sources area of Lancangjiang are in pristine areas, so the majority of SO_4_^2−^ are derived from gypsum dissolution. This result suggests the river water may also need treatment, even with a small amount of anthropogenic input. 

**Figure 7:**
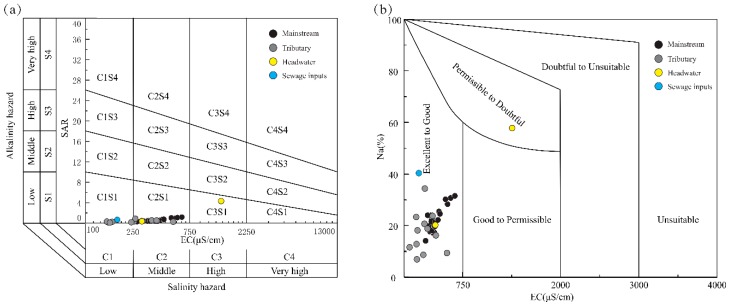
The assessment of irrigation water quality for alkalinity and salinity: (**a**) USSL diagram; (**b**) Wilcox diagram.

The calculation results of the irrigation water quality including SAR, Na% and RSC values are shown in the Table 2. SAR and Na% values represent the sodium hazard to soil aggregates by irrigation; the majority of samples can be defined as the excellent or good quality (SAR < 1; Na% < 30%). The samples in the source area exceed the desirable limits, as the halite dissolution leads to the high SAR and Na% values. RSC as residual sodium carbonate should be lower than 1.25 for irrigation water [52] and the RSC values of all the samples are within the recommended values. Additionally, the USSL and Wilcox diagrams using the EC, Na% and SAR are shown in Figure 7, most of river waters are plotted in the area of C1S1, C2S1 in USSL diagram and in the ‘Excellent to Good’ zone in Wilcox diagram. The samples in the sources area exhibit higher EC values compared to the lower reaches; the sample LCJ-43 is unsuitable to irrigating.

For most sites, the river waters are suitable for drinking purpose and agricultural activities at present and will not lead to health and soil problems in terms of major ions. In the upper reaches of the Lanjiang river, the dissolutions of the evaporite and carbonate transport abundant ions into the river water and as a consequence, the river waters need cleaning before consumption. Thus, the monitoring of water quality should be a long and serious mission. However, all the above water quality assessments are based on major ions to judge whether the river water is suitable to use. The heavy metal elements and organic matters also need to be taken into account, thus more attention should be paid toward these factors in future studies. 

## 4. Conclusions

The spatial distribution of the physicochemical parameters and major ions (Ca^2+^, Mg^2+^, Na^+^, K^+^, Cl^−^, SO_4_^2−^, HCO_3_^−^) were measured. By employing the Pearson correlation coefficients and elemental ratios, the contributions of different sources were determined. The water quality for irrigation and drinking were evaluated. The conclusions can be summarized as follows:

(1) The river water chemistry is controlled by mineral weathering rather than anthropogenic inputs. The concentration order of the anions is HCO_3_^−^ > SO_4_^2−^ > Cl^−^, while the order of cations is Ca^2+^ > Mg^2+^ > Na^+^ + K^+^. Most samples are of the HCO_3_^−^-Ca·Mg type, the river waters are slightly alkaline, and water temperatures decrease as the elevation increases.
(2) The employment of the elemental ratios indicates the mixing relationships between different rocks. The chemical composition of the river water is the mixture of carbonate weathering inputs, evaporite dissolution input and silicate weathering input. In the source area of Lancangjiang River, the complex lithology lead to a relatively large difference in the chemical composition of each tributary.
(3) The forward method indicates that on the watershed scale, the contributions of the atmospheric inputs are limited (<5%), the gypsum dissolution and carbonate minerals dissolution make up ~60% of the total cation budgets (34.9% for carbonate weathering, 24.7% for gypsum dissolution, respectively), the silicate weathering constitutes 17.4% while the halite dissolution makes up 19.2% of the total cations. This result reflects the dissolution rates of different minerals and lithologic distributions, the predominance of the Ca^2+^ and HCO_3_^−^ in river water results from both the wide distribution, and the fast dissolution rate of carbonate minerals
(4) The water quality is generally suitable for irrigation and drinking purposes in terms of major ions. In the upper reaches of Lancangjiang River, the dissolution of gypsum and halite transport abundant Na^+^ and SO_4_^2−^ into river waters, and these contents exceed the recommended values, which may cause health and soil problems, such as soil compaction and salinization. This result suggests even in the pristine areas with little human disturbance, the water quality requires monitoring and assessment due to chemical weathering processes.

## Figures and Tables

**Table 1:** Pearson correlation coefficients between major ions in the Lancangjiang River.

Parameters	DIC	F^−^	Cl^−^	NO_3_^−^	SO_4_^2^^−^	Ca^2+^	K^+^	Mg^2+^	Na^+^	EC
DIC	1									
F^−^	0.643 **	1								
Cl^−^	0.349 *	0.306 *	1							
NO_3_^−^	0.147	0.175	0.142	1						
SO_4_^2^^−^	0.664 **	0.621 ***	0.817 **	−0.006	1					
Ca^2+^	0.839 **	0.731 **	0.731 **	0.055	0.958 **	1				
K^+^	0.337 *	0.391 **	0.693 **	0.683 **	0.531 **	0.507 **	1			
Mg^2+^	0.805 **	0.657 **	0.651 **	0.087	0.939 **	0.959 **	0.414 **	1		
Na^+^	0.428 **	0.365 *	0.995 **	0.172	0.846 **	0.780 **	0.711 **	0.694 **	1	
EC	0.669 **	0.552 **	0.909 **	0.105	0.967 **	0.946 **	0.643 **	0.894 **	0.936 **	1

* Significance at 0.05 probability level; ** Significance at 0.01 probability level.

**Table 2:** The chemical compositions of the river water and water quality guidelines.

Parameters	Min	Max	Mean	SD	Chinese Guidelines	WHO Guideline
pH	7.76	8.77	8.32	0.20	6.5–8.5	6.5–8.5
TDS (mg/L)	52.7	1313	286	189	1000	1000
F^−^ (mg/L)	0.01	0.33	0.16	0.06	1	1.5
Cl^−^ (mg/L)	0.13	331	22.8	48.1	250	250
NO_3_^−^-N (mg/L)	0.05	2.84	0.64	0.44	20 mg/L as N	50
SO_4_^2−^ (mg/L)	3.37	455	88.3	77.4	250	250
SAR	0.08	4.31	0.58	0.61	/	/
Na (%)	7.10	57.9	22.1	8.4	/	/
RSC	−7.04	0.39	−1.18	1.22	/	/

## Supplementary Materials

The following are available online at https://www.mdpi.com/1660-4601/16/23/4670/s1. Table S1: The sampling locations and chemical compositions of the Lancangjiang River, Table S2: Calculation results of forward method and water quality.
